# Intra- and Postoperative Electrocochleography May Be Predictive of Final Electrode Position and Postoperative Hearing Preservation

**DOI:** 10.3389/fnins.2017.00291

**Published:** 2017-05-29

**Authors:** Brendan P. O'Connell, Jourdan T. Holder, Robert T. Dwyer, René H. Gifford, Jack H. Noble, Marc L. Bennett, Alejandro Rivas, George B. Wanna, David S. Haynes, Robert F. Labadie

**Affiliations:** ^1^Department of Otolaryngology, Vanderbilt University Medical CenterNashville, TN, United States; ^2^Department of Hearing and Speech Sciences, Vanderbilt University Medical CenterNashville, TN, United States; ^3^Department of Computer Science and Electrical Engineering, Vanderbilt UniversityNashville, TN, United States

**Keywords:** cochlear implant, electrocochleography, residual hearing, audiometry, cochlear microphonic, hearing loss, hearing preservation

## Abstract

**Introduction:** The objectives of the current study were to (1) determine the relationship between electrocochleography (ECochG), measured from the cochlear implant (CI) electrode array during and after implantation, and postoperative audiometric thresholds, (2) determine the relationship between ECochG amplitude and electrode scalar location determined by computerized tomography (CT); and (3) determine whether changes in cochlear microphonic (CM) amplitude during electrode insertion were associated with postoperative hearing.

**Materials and Methods:** Eighteen subjects undergoing CI with an Advanced Bionics Mid-Scala device were prospectively studied. ECochG responses were recorded using the implant coupled to a custom signal recording unit. ECochG amplitude collected intraoperatively concurrent with CI insertion and at activation was compared with audiometric thresholds postoperatively. Sixteen patients also underwent postoperative CT to determine scalar location and the relationship to ECochG measures and residual hearing.

**Results:** Mean low-frequency pure tone average (LFPTA) increased following surgery by an average of 28 dB (range 8–50). Threshold elevation was significantly greater for electrodes with scalar dislocation. No correlation was found between intraoperative ECochG and postoperative behavioral thresholds collapsed across frequency; however, mean differences in thresholds measured by intraoperative ECochG and postoperative audiometry were significantly smaller for electrodes inserted completely within scala tympani (ST) vs. those translocating from ST to scala vestibuli. A significant correlation was observed between postoperative ECochG thresholds and behavioral thresholds obtained at activation.

**Discussion:** Postoperative audiometry currently serves as a marker for intracochlear trauma though thresholds are not obtained until device activation or later. When measured at the same time-point postoperatively, low-frequency ECochG thresholds correlated with behavioral thresholds. Intraoperative ECochG thresholds, however, did not correlate significantly with postoperative behavioral thresholds suggesting that changes in cochlear physiology occur between electrode insertion and activation. ECochG may hold clinical utility providing surgeons with feedback regarding insertion trauma due to scalar translocation, which may be predictive of postoperative hearing preservation.

**Conclusion:** CI insertion trauma is generally not evident until postoperative audiometry when loss of residual hearing is confirmed. ECochG has potential to provide estimates of trauma during insertion as well as reliable information regarding degree of hearing preservation.

## Introduction

Cochlear implants (CI) are surgically-implanted medical devices capable of restoring audibility and speech understanding to individuals with sensorineural hearing loss (SNHL) who do not receive benefit from appropriately fit amplification. Traditionally, CIs have been used to treat individuals with severe-to-profound hearing loss; however, indications for implantation have expanded to include individuals with significant low-frequency hearing and poor-to-fair speech understanding. Furthermore, advances in electrode design (e.g., increased flexibility and smaller dimensions) and surgical techniques (e.g., surgical approach, insertion angle, insertion speed, etc.) have introduced a new generation of implant recipients with preserved low-frequency hearing in the implanted ear.

The importance of low-frequency hearing in the implanted ear has been well-documented. Preservation of acoustic hearing allows individuals with CIs to take advantage of periodicity, commonly referred to as voice pitch, and temporal fine structure (e.g., Rosen, 1992), offering improved spectral resolution. Periodicity and fine structure provided via residual low-frequency hearing in the implanted ear afford significant improvement for speech understanding in complex listening environments over electric only listening and traditional bimodal hearing combining the CI with acoustic hearing originating from the non-implanted ear (e.g., Dorman and Gifford, 2010; Dunn et al., 2010; Gifford et al., 2013, 2015, 2017; Rader et al., 2013; Loiselle et al., 2016), as well as, significant improvements in sound localization (Dunn et al., 2010; Gifford et al., 2014; Loiselle et al., 2016; Plant and Babic, 2016). The degree of mean hearing preservation benefit ranges from 10- to 20-percentage points for fixed signal-to-noise ratio (SNR) conditions (e.g., Gifford et al., 2013, 2017; Loiselle et al., 2015) and 2–3-dB for adaptive SNR testing (e.g., Dunn et al., 2010; Gifford et al., 2013, 2015). Despite the success of hearing preservation surgery and associated functional benefit, there is still considerable variability in benefit across listeners, and rates of hearing preservation are highly variable across patients, electrode types (perimodiolar and straight), and insertion depths.

Previous studies have demonstrated the benefits associated with low frequency acoustic hearing, but given current resources, surgeons are able to achieve hearing preservation—defined as postoperative audiometric thresholds within 10 dB of preoperative levels—in, at most, 50% of cases (Jurawitz et al., 2014; Santa Maria et al., 2014; Van Abel et al., 2015; Dedhia et al., 2016; Eshraghi et al., 2016; Skarzynski et al., 2016). The pathophysiology of hearing loss during and following surgery is still largely unknown, but it is believed to be a result of (1) intraoperative physical trauma including fracture of the osseous spiral lamina, trans-scalar dislocation, and/or insult to spiral ligament or stria vascularis and/or (2) postoperative inflammatory responses and subsequent fibrosis, neo-osteogenesis and/or cellular apoptosis (e.g., Eshraghi and Van de Water, 2006; Eshraghi et al., 2013; Kamakura and Nadol, 2016).

At present, surgeons and audiologists have no way of knowing whether residual hearing was preserved until the patient returns for audiometric evaluation approximately 2 weeks after surgery. More often than not, there are no indications of physical trauma associated with insertion given the lack of visualization beyond the basal turn. Even experienced surgeons cannot reliably detect the subtle intraoperative forces, which can impart damage to delicate intracochlear structures. Previous retrospective research has shown that the frequent occurrence of translocation from scala tympani (ST) to scala vestibuli (SV) during insertion—occurring in approximately 42% of perimodiolar electrode insertions—has detrimental effects on CI outcomes (Adunka et al., 2004; Finley et al., 2008; Choudhury et al., 2012; Holden et al., 2013; Wanna et al., 2014; Dalbert et al., 2016).

If an intraoperative metric existed that could alert surgeons to physiological damage, such information would potentially allow him/her to modify the surgical procedure and potentially improve outcomes. One emerging solution is the use of intraoperative, intracochlear electrocochleography (ECochG) in providing continuous real-time recordings of physiological activity of intracochlear tissue during and after electrode insertion. ECochG can be recorded for patients with profound hearing loss and even in some individuals with no measurable audiometric thresholds (Choudhury et al., 2012).

ECochG is a technique used to record acoustically evoked electrical potentials generated by the inner ear and auditory nerve. Acoustic stimulation (i.e., a tone burst) is presented to the external ear, and the resulting electrical potentials are measured from the cochlea. The ECochG response is comprised of the cochlear microphonic (CM), summating potential (SP), compound action potential (CAP), and auditory nerve neurophonic (ANN). Each of these responses comes from different parts of the intricate inner auditory system. The CM is thought to represent the electrical potential generated by the stereocilia of the outer hair cells (Sohmer et al., 1980; Patuzzi et al., 1989; Verpy et al., 2008); the SP from the direct current shift of the receptor potential of the inner hair cells and some outer hair cells (Palmer and Russell, 1986; Durrant et al., 1998); the CAP from VIIIth nerve activity (ABR wave I) (Durrant et al., 1998); and the ANN from the inner hair cells (first order generator) and the phase-locked responses of VIIIth nerve fibers, which are used for hearing speech in background noise, localizing sounds, and perceiving/differentiating pitch (Palmer and Russell, 1986; Forgues et al., 2014).

ECochG responses were first recorded using surface electrodes (Poch-Broto et al., 2009), trans-tympanic electrodes (Yoshie et al., 1967; Prijs, 1991; Schoonhoven et al., 1996), or extra-tympanic electrodes (Cullen et al., 1972; Yoshie, 1973; Ferraro, 2010; Zhang, 2012). More recently, potentials have been recorded directly from the cochlea using a needle electrode placed at the round window (Mandala et al., 2012; Radeloff et al., 2012; Dalbert et al., 2015b; Adunka et al., 2016), a needle electrode placed inside the round window (Calloway et al., 2014), or an electrode on the cochlear implant array being implanted (Campbell et al., 2015; Dalbert et al., 2015a).

### Relationship between intraoperative ECochG and postoperative word recognition

Fitzpatrick et al. (2014) recorded ECochG responses at the round window intraoperatively prior to CI insertion in 21 adults and subsequently correlated ECochG magnitude with postoperative CNC word recognition scores. In this study, the metric for ECochG magnitude was termed total response (TR) and defined as the sum of all significant first and second harmonic responses across all frequencies at the highest sound level (90 dB nHL). They reported that TR accounted for 47% of variability in outcomes on the CNC word recognition task making it, at the time, the highest known predictor of CI outcomes even over other predictors such as duration of deafness (<25%; e.g., Rubinstein et al., 1999; Friedland et al., 2003; Plant et al., 2016) and degree of residual hearing (e.g., Plant et al., 2016). Scott et al. (2016) completed intraoperative ECochG with a needle electrode at the round window prior to electrode insertion for 238 CI recipients with postoperative CNC word recognition obtained for 51 adult CI recipients. Similar to Fitzpatrick et al. (2014), they found a significant correlation between TR and CNC word recognition at 6 months post activation (*r* = 0.43); however, the ECochG CAP only weakly correlated postoperative word recognition (*r* = 0.20, *p* < 0.001). Thus, while ECochG appears to be a promising measure for helping explain postoperative outcomes, much additional research is needed to carefully investigate this relationship.

### Relationship between intraoperative ECochG and acoustic hearing preservation

Researchers have also investigated the relationship between intraoperative ECochG and acoustic hearing preservation in the implanted ear. Adunka et al. (2016) recorded ECochG at the round window before and after CI insertion and found no correlation between the ECochG response and postoperative residual hearing as measured by audiometric thresholds—though the results may have been limited by the extracochlear nature of the recording electrode.

ECochG can also be recorded using the CI electrode array which offers advantages given its proximity to the organ of Corti. Koka et al. (2016) measured difference and summation responses from ECochG waveforms postoperatively from patients with residual hearing and compared with behavioral audiometric thresholds. The group found that 87% percent of the variability in postoperative behavioral audiometric thresholds across all frequencies tested could be predicted by difference response thresholds and 82% predicted by summation response thresholds; concluding that ECochG thresholds may be useful to estimate postoperative preserved acoustic hearing in CI patients who cannot participate in behavioral audiometry.

Campbell et al. (2016) recorded ECochG measurements intraoperatively from the CI array in 18 recipients with residual acoustic hearing and (1) explored providing real-time surgical feedback as well as (2) investigated the correlation between ECochG recordings and postoperative acoustic hearing. They found this method to be potentially useful for providing feedback regarding surgical trauma and that patients who had a preserved ECochG at the end of surgery were more likely to have preserved hearing. In fact, postoperative audiometric thresholds for patients with preserved CM were, on average, 15 dB better than individuals without a preserved ECochG. Similar findings were reported by Acharya et al. (2016) for two pediatric patients.

Building on this previous work, in the present study intracochlear ECochG responses were measured for 18 (*n* = 18) adult Advanced Bionics (AB) CI recipients with preoperative acoustic hearing in the ear to be implanted. ECochG measurements were made both during and after CI insertion, and these measures were compared with pre- and postoperative audiometric thresholds. Sixteen (*n* = 16) participants also underwent postoperative computerized tomography (CT) scanning to verify scalar placement. The objectives of the current study were (1) to determine the relationship between ECochG, measured from the CI array either during cochlear implantation or after surgery, and postoperative audiometric thresholds, (2) to determine if the CM amplitudes correlated with electrode scalar location/translocation as determined by CT scanning, and (3) to determine if change in CM during electrode insertion is associated with postoperative residual hearing.

## Methods

### Subjects

Adult patients with residual acoustic hearing (≤80 dB HL at 250 Hz) who were seeking cochlear implantation with an Advanced Bionics (AB) Mid-Scala device between April and December 2016 were prospectively recruited for participation. Exclusion criteria included previous history of middle ear surgery, sudden sensorineural hearing loss, auditory neuropathy spectrum disorder (ANSD), single-sided deafness, and/or abnormal anatomy as detected by CT or MRI scanning. Eighteen (*n* = 18) subjects met inclusion criteria and were implanted by one of five cochlear implant surgeons using a round window (*n* = 14) or extended round window approach (*n* = 4). Patient demographics are shown in Table 1. The methods used in this study were in accordance with the ethical standards of the institutional review board at Vanderbilt University (IRB approval: 151808), and all subjects provided written informed consent before participation.

**Table 1:** **Subject demographics, RW, round window; ERW, extended round window; LFPTA, low frequency pure tone average (average threshold for 125, 250, and 500 Hz, in dB HL); ST, scala tympani; SV, scala vestibuli; Preop, preoperative; Postop, postoperative**.

**Subject**	**Surgical approach**	**Preop LFPTA**	**Postop LFPTA**	**LFPTA shift**	**Scalar location**
1	ERW	50.0	61.7	11.7	ST
2	ERW	51.7	85.0	33.3	ST-SV
3	RW	60.0	105.0^*^	45.0	ST-SV
4	RW	68.3	88.3	20.0	ST
5	RW	63.3	105.0^*^	41.7	ST-SV
6	RW	41.7	76.7	35.0	ST-SV
7	RW	31.7	81.7	50.0	–
8	ERW	31.7	56.7	25.0	ST
9	RW	56.7	105.0^*^	48.3	ST-SV
10	RW	66.7	105.0^*^	38.3	ST (^*^ BM)
11	RW	66.7	76.7	10.0	ST-SV
12	RW	26.7	45.0	18.3	ST
13	RW	45.0	70.0	25.0	ST
14	ERW	53.3	66.7	13.3	ST
15	RW	58.3	105.0^*^	46.7	–
16	RW	75.3	83.3	8.0	ST
17	RW	66.7	75.0	8.3	ST
18	RW	60.0	80.0	20.0	ST
**MEAN**	**–**	**54.1**	**81.8**	**27.7**	**–**

Thresholds with asterisk represent no behavioral response at the limits of the audiometer.

* *BM indicates the electrode pushing against the basilar membrane*.

### Equipment

The equipment used for data collection was previously described by Koka et al. (2016). The Bionic Ear Data Collection System (BEDCS) was used to measure ECochG responses. A NI DAQ system (NI DAQ 6216, National Instruments Corporation, 11500 Mopac Expwy, Austin, TX) and an audio amplifier (Sony PHA-2, Sony Corporation, New York, NY) were used to generate the acoustic stimuli, which was presented through an ER-3A (Etymotic Research, Inc. 61 Martin Lane, Elk Grove Village, IL) insert earphone. An ER-7 (Etymotic Research, Inc. 61 Martin Lane, Elk Grove Village, IL) probe microphone was used to calibrate and monitor the stimulus level in the ear canal. The ECochG response was measured using an AB Clinical Programming Interface Platinum Series Sound Processor (PSP) and Universal Headpiece (UHP) with additional magnets for retention and secure connection.

### Pure-tone audiometry (PTA)

Pure-tone audiometry was assessed prior to implantation and at activation approximately 2–3 weeks after surgery. Audiometric thresholds were completed in a double-walled sound treated booth. Air-conduction thresholds were obtained for all octaves and inter-octave frequencies from 125 to 8,000 Hz using an insert earphone. Bone-conduction thresholds were obtained for octave frequencies from 500 to 4,000 Hz using a bone oscillator placed on the mastoid. Contralateral masking was implemented when appropriate. Low-frequency PTA was calculated using the average of unaided air-conduction thresholds at 125, 250, and 500 Hz.

### ECochG recording

ECochG potentials were measured from the most apical electrode of the implant array intraoperatively as the surgeon was inserting the CI and postoperatively at each subject's CI activation. Intraoperatively, after the patient was intubated, an ER-3A (Etymotic Research, Inc. 61 Martin Lane, Elk Grove Village, IL) insert earphone and an ER-7 (Etymotic Research, Inc. 61 Martin Lane, Elk Grove Village, IL) probe microphone were placed in the external auditory canal of the surgical ear (See Koka et al., 2016, Figure 1). Since the insert earphone and probe microphone were not sterilized, these pieces were kept out of the sterile field by folding the pinna anteriorly and securing it with a large Tegaderm® transparent adhesive film dressing (3M, 2501 Hudson Rd., Maplewood, MN) taking caution to not compromise the tube delivering sound to the ear. At this point, calibration was completed to ensure that the tube was not crimped or that the insert placement was faulty. The cables/tubes connecting the insert earphone and probe microphone to the measurement equipment were then disconnected, wrapped in a cloth, and placed underneath the surgical table so as to minimize interference with the surgical procedure. The surgical preparation (i.e., sterilization and draping) and surgical procedure (cortical mastoidectomy, facial recess, and round window exposure) then progressed according to normal protocols until just before insertion of the electrode array at which point the cables/tubes were reconnected to the recording equipment and the Universal Headpiece and cable were covered with a sterile ultrasound bag and magnetically coupled to the patient's newly implanted receiver/stimulator. Calibration was repeated, and the ECochG recording was started. The CI electrode was introduced via the round window or extended round window and inserted according to the manufacturer's recommendations (i.e., insertion with the stylet to the first blue marker at which point the pre-curved electrode was advanced off the stylet until the second blue marker was located at the round window). The surgeon reported a full insertion in all cases. While the surgeon was inserting the electrode, the audiologist used markers to identify different key points during the surgery (i.e., round window, first blue marker, second blue marker, complete insertion). For the duration of electrode insertion and ECochG insertion, an acoustic tone burst was delivered via the insert earphone (500-Hz, toneburst, 110 dB SPL or 97 dB HL, alternating polarity, 50-ms duration with 5-ms onset/offset ramp time) while the ECochG response was recorded from the most apical electrode. The neural response imaging (NRI) amplifier in the implant was used for amplification of the response (gain of 1,000). The recordings were done with alternating polarities (2 rarefaction and 2 condensation traces) and averaged in the implant amplifier, then transferred to the processor. Data plotting for the insertion tracks depends on SNR of the signal, which usually averages and plots at a single point until SNR reaches 18 dB, or 8 averages have been performed (internally 16 averages). The SNR benefit can be achieved by 55 ms recordings that can be seen in frequency spectrum with larger acquisition times; the acquisitions were done at 4–6 stimuli per second. In presenting this data, the CM amplitude during the insertion track is normalized with respect to the amplitude obtained at the round window, therefore values are presented as dB. After insertion was complete, the recording electrode was changed to 1, 5, 9, and then 13; additional ECochG measurements were obtained from these electrodes to try and understand electrode location with respect to the 500-Hz stimulus. Subsequently, the stimulus frequency was changed from 125 to 2,000 Hz in octave steps using electrode 1 as the recording electrode to estimate each subject's CM threshold in dB HL at each frequency. Surgery concluded per standard. It is estimated that intraoperative ECochG testing added approximately 5 min of time to each case. It should be noted that for this study, the surgeon was not informed of the ECochG results during the insertion of the electrode.

**Figure 1:**
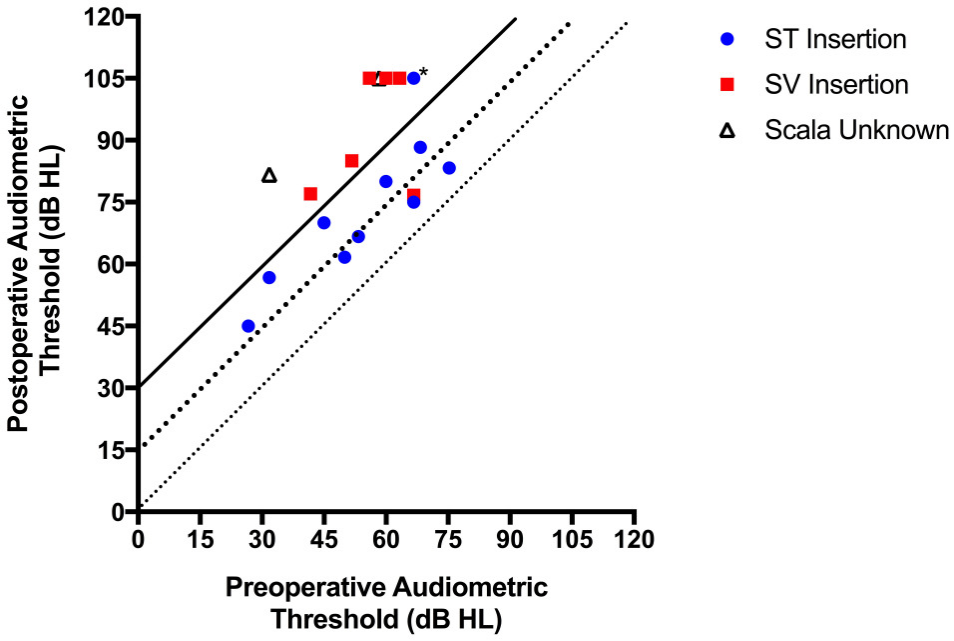
**Pre- and postoperative pure-tone thresholds; each symbol represents an individual patient**. Scalar location of electrode, when available, has been denoted (the ^*^ represents the electrode abutting the basilar membrane). Diagonal lines are used to depict hearing preservation in relation to pure-tone average (PTA) shift bins as follows: PTA shift <15 dB, PTA shift between 15–30 dB, and PTA shift >30 dB.

Postoperative ECochG measurement occurred in the audiology clinic on the same day as the patient's CI activation appointment, typically 2 weeks after surgery. An ER-3A (Etymotic Research, Inc. 61 Martin Lane, Elk Grove Village, IL) insert earphone and an ER-7 (Etymotic Research, Inc. 61 Martin Lane, Elk Grove Village, IL) probe microphone were placed in the external auditory canal of the implanted ear, and the Universal Headpiece was coupled with the patient's receiver stimulator. Calibration was completed to ensure that the tube was not crimped or that the insert placement was faulty. Tone bursts were presented sequentially at 125, 250, 500, 1,000, and 2,000 Hz. The patient's ECochG response was measured from the apical electrode and recorded for each frequency. These frequency scan responses were used to estimate subjects' CM thresholds.

### Stimuli and recording parameters

The amplitude of the ECochG response was calculated using fast Fourier transformation (FFT) analysis within the Bionic Ear Data Collection System. A sample rate of 9,280 and a low pass filter of 5 kHz in the NRI amplifier were used to acquire the responses over a 54.5 ms recording duration through back-telemetry.

### Computerized tomography (CT) scanning

A subset (*n* = 16) of patients received postoperative CT scans using a low-dose, flat-panel, volumetric computerized tomography machine (Xoran XCAT, Xoran Technologies; Ann Arbor, MI). Using previously described and validated image-processing algorithms (Noble et al., 2011) scans were analyzed for scalar location of the electrode array (Noble et al., 2011). ST insertions were defined as insertions in which all electrode contacts were located entirely within the ST. Conversely, SV insertions were characterized by electrode arrays that translocated from the ST into the SV, such that at least one electrode contact was located within the SV.

### Statistical methods

Data were plotted and analyzed using GraphPad Prism 7.0 software (GraphPad Software Inc, 2012). Continuous variables were tested for normal distribution with D'Agostino and Pearson omnibus normality test.

Correlations were performed to examine the relationships between ECochG thresholds and behavioral thresholds at individual frequencies (125, 250, and 500 Hz). Parametric and nonparametric data were examined using a Pearson or Spearman correlation analysis, respectively. Spearman correlation was also used if the sample size of a group was too small to determine distribution of data. Given that correlations were performed at multiple frequencies, the Bonferroni correction was used adjusting the critical *p*-value. Patients were then categorized by the scalar location of their electrode array (ST and SV), and correlations between ECochG and behavioral thresholds within both these groups were assessed.

The following dependent variables were also assessed: (1) the absolute difference between ECochG thresholds and behavioral thresholds at individual frequencies (125, 250, and 500 Hz), (2) low-frequency PTA shift, (3) rise in CM amplitude from start of insertion to the peak value during insertion, and (4) the drop in CM amplitude from the peak value during insertion to completion of insertion. Patients were again characterized into groups according to scalar location and comparisons of the aforementioned variables were made between ST vs. SV insertions with an independent *t*-test (normal distribution) or a Mann-Whitney *U-*test (non-normal distribution). A *p* < 0.05 was considered indicative of statistical significance, with the exception of data pertaining to absolute differences between ECochG thresholds and behavioral thresholds, as multiple frequencies were analyzed; the Bonferroni correction was used in these analyses.

## Results

### Demographics and operative characteristics

Eighteen patients met inclusion criteria and were prospectively enrolled (Table 1). The median age at the time of surgery was 67 years (range 23–80); 61% of the patients were male. Round window insertions were performed in the 78% of cases (*n* = 14), with extended round window insertions used in the remaining 22% (*n* = 4). Surgeons reported full insertion in all cases. Resistance during insertion was subjectively noted in one case; with electrode repositioning resistance subsided and a full insertion was achieved.

### Electrode location

Sixteen patients consented to undergo postoperative CT imaging such that scalar electrode location could be determined. Two patients electively chose not to participate in the postoperative imaging portion of the study, therefore scalar location of these electrode arrays could not be determined. Because all insertions were performed through either round window or extended round window approaches, all electrodes were initially inserted into the ST within the basal turn. In six patients (38%), electrode translocation from the ST into the SV was observed. In one patient, after analysis, the electrode array was pushing against the basilar membrane but did not clearly translocate into the SV; interestingly, this was the case in which resistance was subjectively felt during insertion. Because of the limits of our image processing algorithms, this patient was excluded from subsequent statistical analyses that examined the impact of scalar location on audiologic outcomes.

### Hearing preservation

Preoperatively, all patients had functional residual hearing (≤80 dB HL at 250 Hz) prior to surgery. The mean preoperative low-frequency PTA was 54 dB HL (range 27–75). At activation, the majority of patients (*n* = 12, 66%) demonstrated measurable unaided air-conduction thresholds at 125, 250, and 500 Hz. One patient had measurable thresholds at 125, and 250 Hz but did not respond to unaided pure-tones at 500 Hz; the remaining 5 patients demonstrated no responses at 125, 250, and 500 Hz.

Eleven patients (61%) maintained thresholds ≤80 dB HL at 250 Hz. Mean low-frequency PTA at activation was 82 dB HL (range 45–105), yielding an average low-frequency PTA shift of 28 dB (range 8–50). As depicted in Figure 1, 5 patients (28%) demonstrated low-frequency PTA shift <15 dB, 5 patients (28%) demonstrated low-frequency PTA shift between 15 and 30 dB, and the remaining 8 patients demonstrated low-frequency PTA shift >30 dB (44%).

**Figure 2:**
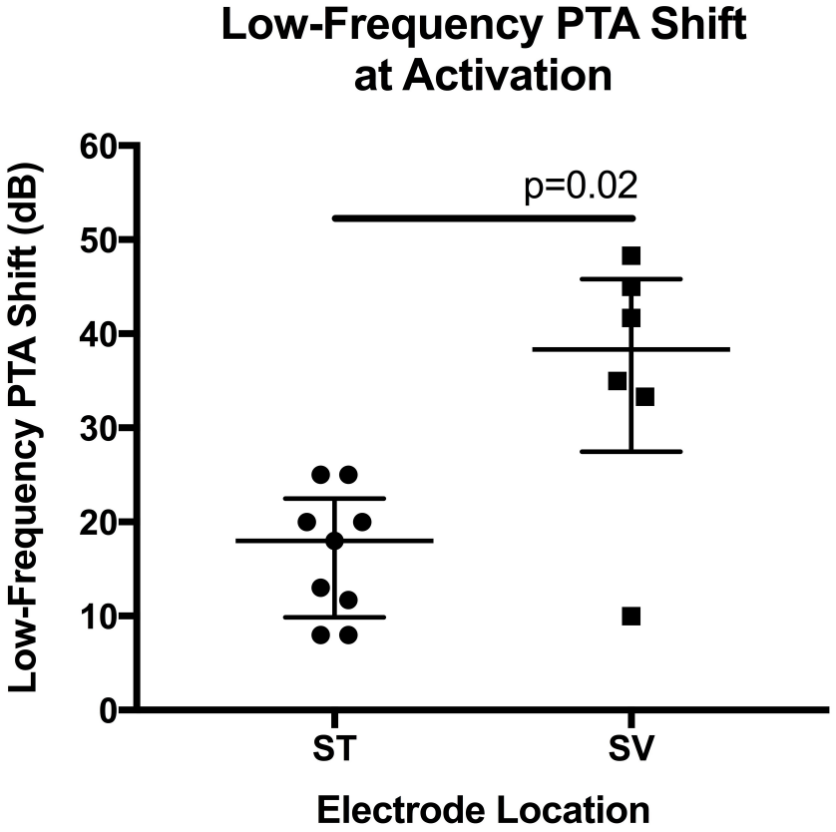
**Scatter plot of low-frequency pure-tone average (PTA) shift depicted according to scalar electrode location**. Lower median shift (i.e., better hearing preservation) was noted when comparing electrodes inserted entirely into the scala tympani (ST) to electrodes that translocated into scala vestibuli (SV). Shown are the median and the range of the 25–75th percentile.

The impact of demographic and surgical variables on low-frequency PTA shift was then assessed. No relation between age at surgery and postoperative PTA shift was noted (*r* = 0.13, *p* = 0.60). Further, no difference in median PTA shift was observed when round window insertions (23 dB, range 8–50) were compared to extended round window insertions (22 dB, range 12–47, *p* = 0.81). The median low-frequency PTA shift was significantly lower for electrodes entirely inserted into the ST (16 dB, range 8–25) as compared to electrodes that translocated into the SV (38 dB, range 10–48, *p* = 0.02; Figure 2).

### Intraoperative ECochG thresholds vs. postoperative behavioral thresholds

**Figure 3:**
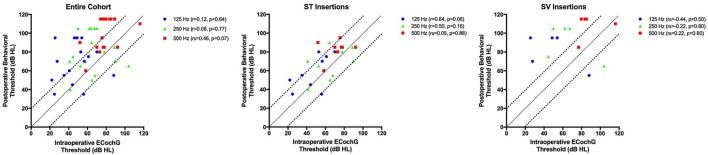
**The relationship between intraoperative ECochG thresholds, in dB HL, and postoperative behavioral thresholds, also in dB HL, for 125, 250, and 500 Hz are depicted in the entire cohort, and for those cases in which scalar location is known**. Bonferroni correction is applied for multiple comparisons, with *p* < 0.017 indicative of statistical significance. The diagonal and dotted lines represent the ±20 dB difference between ECochG thresholds and behavioral thresholds.

The relationship between intraoperative ECochG thresholds and postoperative behavioral audiometric thresholds was analyzed. Intraoperative ECochG thresholds were successfully measured in 17 patients (94.4%); connection between the receiver stimulator and external monitoring equipment was lost in one patient. The absolute mean difference between intraoperative ECochG thresholds and postoperative behavioral thresholds for 125, 250, and 500 Hz is shown in Table 2. The absolute difference between intraoperative ECochG thresholds and postoperative audiometric thresholds was significantly lower (i.e., better) for ST insertions compared to SV insertions at 125 and 250 Hz frequencies (*p* = 0.001 for both analyses). In the overall cohort, no significant correlations between intraoperative ECochG thresholds and postoperative behavioral thresholds were noted at 125 Hz (*r* = 0.12, *p* = 0.64), 250 Hz (*r* = 0.08, *p* = 0.77), or 500 Hz (*r*s = 0.46, *p* = 0.07; Figure 3). The relationship between ECochG and behavioral thresholds at activation is also plotted as a function of scalar location.

**Table 2:** **The mean absolute difference between intraoperative electrocochleography (ECochG) thresholds and postoperative behavioral thresholds at 125, 250, and 500 Hz frequencies are shown in the overall cohort**.

**Frequency (Hz)**	**Δ Intraop ECochG and postop behavioral thresholds, overall mean in dB HL (range)**	**Δ Intraop ECochG and postop behavioral thresholds, ST Insertion mean in dB HL (range)**	**Δ Intraop ECochG and postop behavioral thresholds, SV insertion mean in dB HL (range)**	***p*-value**
125	29 (1–69)	16 (1–28)	46 (33–69)	0.001
250	24 (2–55)	13 (2–29)	41 (30–55)	0.001
500	19 (2–40)	12 (2–38)	22 (6–35)	0.310

*Differences are also depicted according to scalar location of the electrode array; the P value represents the comparison between scala tympani (ST) insertions and scala vestibuli (SV) insertions. Means and ranges are reported. Bonferroni correction is applied for multiple comparisons, with p < 0.017 indicative of statistical significance*.

### Postoperative ECochG thresholds vs. postoperative behavioral thresholds

**Figure 4:**
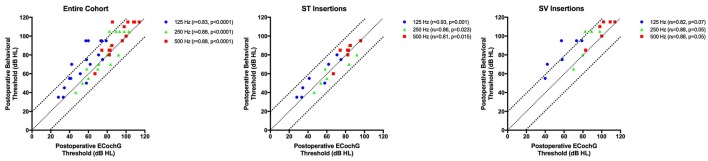
**The relationship between postoperative ECochG thresholds and postoperative behavioral thresholds for 125, 250, and 500 Hz frequencies are depicted in the entire cohort, and for those cases in which scalar location is known**. Bonferroni correction is applied for multiple comparisons, with *p* < 0.017 indicative of statistical significance.

**Figure 5:**
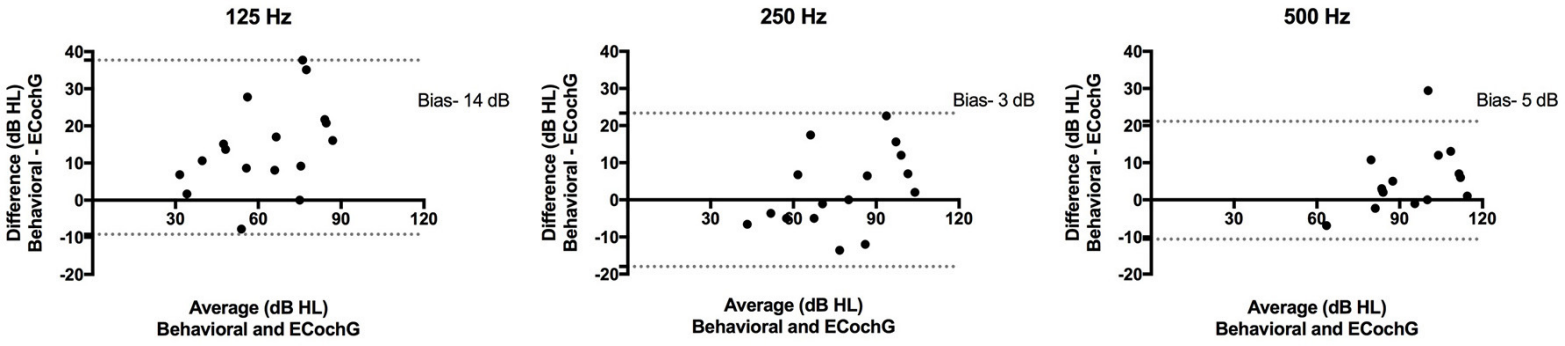
**Bland-Altman plots depict the average and difference between postoperative behavioral and ECochG thresholds at 125, 250, and 500 Hz**. The 95% limits of agreement are shown as two dotted lines. The biases, or average of the differences at each frequency, are reported.

Postoperative ECochG thresholds were successfully measured in 17 patients (94%) at activation; testing in one patient was limited by time constraints and patient preference. The mean difference between ECochG thresholds and behavioral thresholds at activation is shown in Table 3. At 125 Hz, the difference between postoperative ECochG threshold and pure tone thresholds was significantly lower (i.e., better) for ST insertions compared to SV insertions (*p* = 0.0007). A significant correlation between ECochG thresholds and behavioral thresholds at activation was observed at 125 Hz (*r* = 0.83, *p* < 0.0001), 250 Hz (*r* = 0.88, *p* < 0.0001), and 500 Hz (*r* = 0.88, *p* < 0.0001; Figure 4). These relationships are also shown according to scalar location. Bland-Altman plots assessing agreement between methods at activation for low-frequencies are shown in Figure 5.

**Table 3:** **The mean absolute difference between postoperative electrocochleography (ECochG) thresholds and postoperative behavioral thresholds at 125, 250, and 500 Hz frequencies are shown in the overall cohort**.

**Frequency (Hz)**	**Δ Postop ECochG and postop behavioral thresholds, overall mean in dB HL (range)**	**Δ Postop ECochG and postop behavioral thresholds, ST insertion mean in dB HL (range)**	**Δ Postop ECochG and postop behavioral thresholds, SV insertion mean in dB HL (range)**	***p*-value**
125	15 (0–37)	7 (0–14)	22 (15–37)	0.0007
250	9 (0–23)	8 (4–14)	11 (0–23)	0.42
500	6 (0–29)	4 (1–11)	6 (0–13)	0.99

*Differences are also depicted according to scalar location of the electrode array; the P-value represents the comparison between scala tympani (ST) insertions and scala vestibuli (SV) insertions. Means and ranges are reported. Bonferroni correction is applied for multiple comparisons, with p < 0.017 indicative of statistical significance*.

### ECochG insertion monitoring

**Figure 6:**
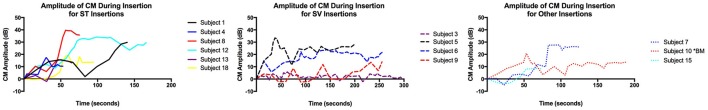
**Change in cochlear microphonic (CM) amplitude, in dB re: microVolts, during insertion is shown according to scalar location of the electrode array**.

Changes in CM amplitude during electrode insertion were then analyzed. As mentioned previously, intraoperative ECochG could not be performed in one patient; in addition, the insertion scans from four other patients were invalid secondary to monitoring issues. Insertion scans from the remaining 13 patients are depicted in Figure 6 according to scalar electrode location. The mean rise in CM amplitude from start of insertion at the round window to the peak value during insertion, was 22 dB (range 5–40). On average, the CM amplitude dropped 3 dB (range 0–8) from the peak value during insertion to completion of insertion. These objective measures of CM amplitude change were compared between ST and SV insertions; no significant differences were noted (*p* = 0.35 and *p* = 0.61; Table 4). Further, low-frequency PTA shift did not correlate significantly with round window to peak amplitude (*r* = −0.40, *p* = 0.17) nor drop from peak to completion of insertion (*r* = 0.26, *p* = 0.38).

**Table 4:** **Various objective measures of change in cochlear microphonic (CM) amplitude during insertion are compared between scala tympani (ST) and scala vestibuli (SV) insertions**.

**Δ CM Amplitude during insertion**	**ST Insertion median in dB (range)**	**SV Insertion median in dB (range)**	***p*-value**
Round window to peak amplitude	25 (16–40)	19 (5–33)	0.35
Peak amplitude to complete insertion	5 (0–8)	3 (0–5)	0.61

## Discussion

In the current study, we completed ECochG obtaining CM amplitude at various stages in the electrode insertion as well as an estimate obtained at the activation appointment. We did not observe a significant relationship between CM amplitude obtained during electrode insertion and scalar electrode location for our group of 16 patients with postoperative CT scans. Intraoperative ECochG thresholds, via frequency scan, did not correlate significantly with postoperative audiometric thresholds; however, a trend was noted between ECochG thresholds and behavioral thresholds for electrodes inserted entirely into the ST at 125 Hz (*p* = 0.06). Further, the mean difference between intraoperative ECochG thresholds and postoperative audiometric thresholds was significantly smaller for electrodes in ST as compared to those which translocated into SV at 125 and 250 Hz.

At present, postoperative audiometric thresholds represent a marker for intracochlear insertion trauma. We hypothesize that intraoperative ECochG may provide us with valuable information at the time of surgery that may be significantly correlated with behavioral audiometric thresholds obtained at activation *if electrodes remain within ST*. Though we did not observe a significant correlation between ECochG thresholds obtained intraoperatively (measured via frequency scan immediately after insertion) and postoperative audiometric thresholds at activation, the difference between intraoperative ECochG thresholds and postoperative audiometric thresholds was *significantly lower* (i.e., better) for electrodes completely located in ST. These data support the notion that changes in cochlear physiology occur in the time period between electrode insertion and activation, and are more pronounced for electrodes that translocate into the SV. Further, these data suggest that ECochG may hold clinical utility providing surgeons with feedback regarding insertion trauma as well as information regarding expected hearing preservation. Additional data are needed with larger sample sizes and broader distributions of preoperative audiometric thresholds in the low-frequency region to thoroughly investigate this relationship.

We also sought to examine whether various objective measures of CM amplitude during electrode insertion (measured via insertion scan) were related to either scalar location or hearing preservation outcomes. In order to objectively assess this relationship, we chose to record the following: (1) rise in CM amplitude from start of insertion at the round window to the peak value during insertion, and (2) drop in CM from the peak value during insertion to completion of insertion. Neither of these measures was found to be associated with scalar location or hearing preservation. It is possible that the small sample size of adequate insertion scans (*n* = 13) limited our analysis in this regard. Alternatively, we may have chosen outcomes measures that lack sensitivity to pick up differences between groups. Further studies assessing amplitude and phase characteristics of the ECochG waveform are warranted. It should be emphasized that no feedback was provided to the surgeon in the current study; we do however, plan to commence a thorough study of the utility of intraoperative ECochG in helping to guide surgical insertion. Should ECochG data obtained during insertion serve as a tool guiding surgical insertion, such feedback may allow for surgical modifications (e.g., redirecting insertion vector) resulting in less traumatic insertions, preservation of intracochlear structures, and potentially, higher rates of hearing preservation.

Current clinical practice uses audiometric thresholds (e.g., Carlson et al., 2011; Cosetti et al., 2013; Sweeney et al., 2016) and retained unaided word recognition in the postoperative period as markers of surgical trauma (inflammation, fibrosis, and/or bone growth). Postoperative audiograms, however, provide only a gross estimate of peripheral auditory function. Furthermore, in standard clinical practice, postoperative acoustic word recognition is rarely obtained for the implanted ear. In some cases, preoperative acoustic word recognition is near zero, rendering retention of word recognition potentially an irrelevant measure. Despite these challenges, the biggest restriction in our current clinical practice is that we are currently unable to assess the effects of implantation trauma until the damage has occurred which is likely irreversible. Thus, we need a measure capable of providing real-time estimates of insertion trauma providing feedback to surgeons during electrode insertion. Theoretically speaking, reducing insertion trauma will potentially result in less fibrosis, bony growth, and cellular apoptosis—though the patient-specific inflammatory response remains an unknown variable. Additional value from such a measure of insertion trauma may help guide clinical decision making regarding administration of postoperative steroids in cases where concerns may arise regarding acoustic hearing preservation.

In addition to investigating the effect of cochlear implantation on ECochG responses measured during surgical insertion, ECochG responses at postoperative activation were also assessed. Significant correlations between postoperative ECochG thresholds and pure-tone behavioral thresholds were noted across low frequencies. Our findings corroborate data recently published by Koka et al. (2016), in which strong agreement between postoperative ECochG thresholds and behavioral thresholds was also demonstrated. As physiologic estimates of hearing thresholds (via ECochG frequency scan) and behavioral measurements of hearing (pure-tone audiometry) correlate well when measured at the same time-point, the fact that intraoperative ECochG thresholds did not correlate with postoperative behavioral hearing herein further supports that cochlear physiology changes in the time between electrode insertion and activation. Future studies examining the differential changes that result directly from electrode insertion vs. those that occur in the acute post-insertion period are needed; controlling for scalar location in such reports appears to be very important. Taken together, ECochG thresholds may be capable of quantifying the degree on insertion trauma and resultant intracochlear physiological changes impacting behavioral hearing thresholds. Lastly, our data may also hold significant clinical value for patients unable to provide reliable behavioral data at the activation appointment and even possibly at subsequent postoperative audiology appointments.

### Limitations

The primary limitation of the current study was the sample size (*n* = 18) and as a result, generalizations cannot be made at this time. Further, though ECochG including CM peak amplitude with electrode insertion may hold future surgical value regarding insertion trauma, no feedback was provided to the surgeons during the insertions on any of the cases included here. In order to thoroughly investigate the utility of this measure—particularly in helping to avoid scalar dislocation—real-time feedback is likely a necessary component. Finally, all participants in the current study were recipients of a conventional, pre-curved electrode, the AB mid-scala electrode. That is, none of the subjects were implanted with a lateral-wall electrode *specifically designed* for hearing preservation. Thus, it is possible that ECochG thresholds may not generalize to recipients of a shorter, lateral-wall electrode who may have lower, and potentially better, audiometric thresholds across a broader range of frequencies. Our research team is actively involved in ongoing efforts to investigate the clinical utility of ECochG as both a measure of intracochlear insertion trauma and postoperative audiometric thresholds in larger sample sizes with patients of varying residual hearing in the low-frequency and both pre-curved and lateral-wall electrodes.

### Summary

More patients are presenting for CI who have measureable and clinically significant preoperative hearing thresholds. However, we are unable to appreciate the effects of CI insertion trauma and resultant postoperative audiometric thresholds until the point of device activation or even later when behavioral hearing thresholds are measured. The current study investigated the relationship between intraoperative and postoperative ECochG measurements and postoperative audiometry in a group of 18 patients with preoperative 250-Hz thresholds up to 80 dB HL who were implanted with an AB mid-scala electrode. Sixteen of the 18 patient consented to postoperative CT imaging allowing for determination of electrode scalar location. From the current dataset, the primary conclusions were as follows:

- Scalar translocation from ST to SV was associated with significantly higher shifts in low-frequency PTA when compared to electrodes inserted entirely within ST.
- There was no statistically significant relationship between intraoperative ECochG thresholds and postoperative audiometric thresholds at the group level.
  - However, a trend was noted between intraoperative ECochG thresholds and postoperative audiometric thresholds when excluding patients for whom electrode crossed from ST to SV.
  - Further, the difference between intraoperative ECochG thresholds and postoperative audiometric thresholds was *significantly lower* (i.e., better) for electrodes completely located in ST.
  - This leads us to conclude that ECochG may hold clinical utility providing surgeons with intraoperative feedback regarding insertion trauma as well as information regarding expected hearing preservation.
- There was a significant relationship between postoperative ECochG thresholds and postoperative audiometric thresholds.
  - This measure may hold significant clinical value for patients unable to provide reliable behavioral data at the activation appointment (e.g., young children) and potentially for appointments when time does not allow for comprehensive device programming and behavioral audiometry.
  - Further this suggests that changes in cochlear physiology following cochlear implantation may be evidenced by changes noted in ECochG data obtained intraoperatively and at various postoperative time points.

## Author contributions

JH, BO, RD, RG, JN, and RL all collaborated on experimental design, data analysis, and manuscript preparation. JH, RD, BO, RL, and RG recruited participants and collected data. BO and RL organized the results and conducted statistical analyses. RL was responsible for the supervision of the operating room methods and CT imaging. JN completed analyses of pre- and post-implant CT imaging. MB, AR, RL, DH, and GW inserted electrode arrays used for data collection. RL and RG supervised the project, secured funding, and provided guidance for methodology and interpretation of findings.

### Conflict of interest statement

RG is on the audiology advisory board for Advanced Bionics and Cochlear Americas and the clinical advisory board for Frequency Therapeutics. RL is a consultant for Advanced Bionics, Cochlear Americas, and Ototronix. DH is on the surgical advisory boards for Cochlear, MED-EL, AB, Stryker, Anspach, and Oticon Medical. MB is on the surgical advisory board for MED-EL and is a consultant for Oticon Medical. AR is on the surgical advisory boards for Cochlear, MED-EL, AB, Stryker, Olympus, and Grace Medical. GW is on the surgical advisory board for Oticon Medical and is a consultant for AB, Cochlear, and MED-EL. The other authors declare that the research was conducted in the absence of any commercial or financial relationships that directly affected the current research.
